# Damage control surgery for life-threatening blunt chest trauma

**DOI:** 10.1093/jscr/rjae449

**Published:** 2024-07-30

**Authors:** Yeon Soo Kim

**Affiliations:** Department of Thoracic and Cardiovascular Surgery, Inje University Ilsan Paik Hospital, 170 Juwha-ro, Ilsansu-gu, Goyang-si 10394, Gyeonsgi-Do, South Korea

**Keywords:** chest trauma, damage control surgery, extracorporeal membrane oxygenation

## Abstract

A 31-year-old male sustained life-threatening chest contusions and recovered after damage control surgery. The patient was in an unwitnessed accident where his motorcycle was struck by a car. Upon admission, blood pressure was 69/58 mmHg, heart rate was 126 bpm, and oxygen saturation was 85%. Chest computed tomography revealed fractures to right ribs 1–9 and left ribs 1–7, lung contusions, multiple lung lacerations, and right hemopneumothorax. Upon presentation to the intensive care unit, hemostasis was achieved by suturing a deeply lacerated lung and applying gauze packing. The patient was placed on veno-veno type extracorporeal membrane oxygenation using both femoral veins after surgery until the 5th hospital day. The gauze was removed during the second operation on the 6th day. The third operation on the 13th hospital day was an open reduction of ribs 3–7 on the right. The patient was discharged on the 47th day without complications.

## Introduction

Damage control surgery (DCS) is performed for initial control of hemorrhage and contamination, followed by physiological recovery in an intensive care unit and then planned definitive surgery. This surgery minimizes definitive operation time and increases the effectiveness of initial management of exsanguinated patients. To broaden its range of application, this concept, which originated in the field of abdominal surgery, has been applied to physiologically exhausted patients in shock and to patients with thoracic injuries [1].

This case report is a patient who visited the hospital in shock due to severe blunt chest trauma and was successfully treated with DCS, intrathoracic gauze packing, and extracorporeal membrane oxygenation (ECMO).

## Case presentation

**Figure 1:**
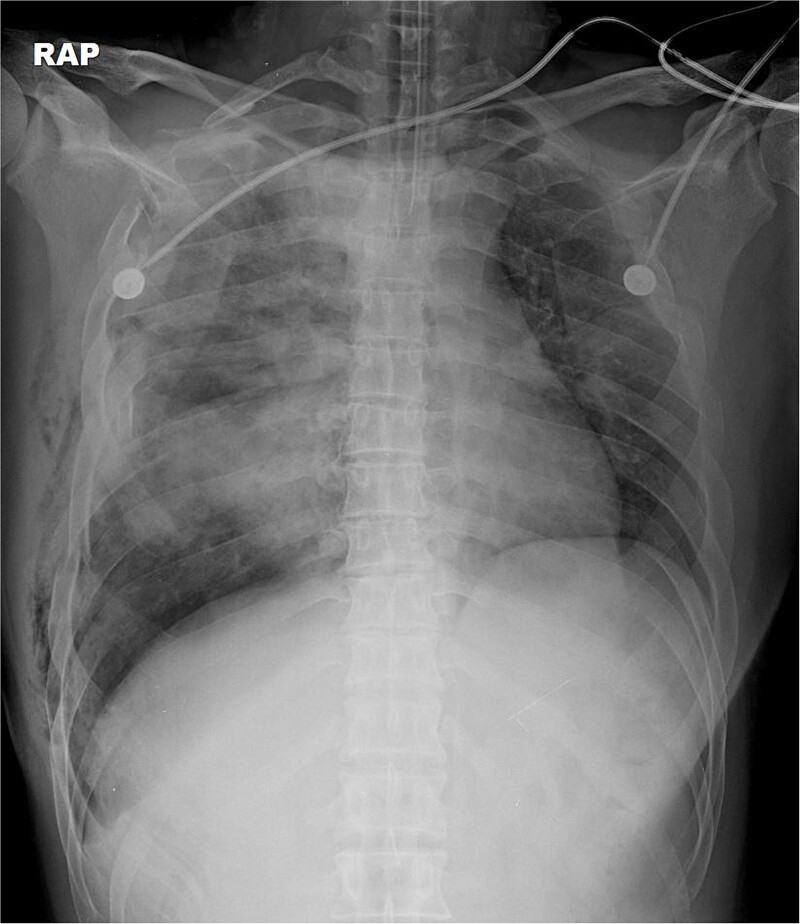
A simple radiograph of a 31-year-old man after injury.

**Figure 2:**
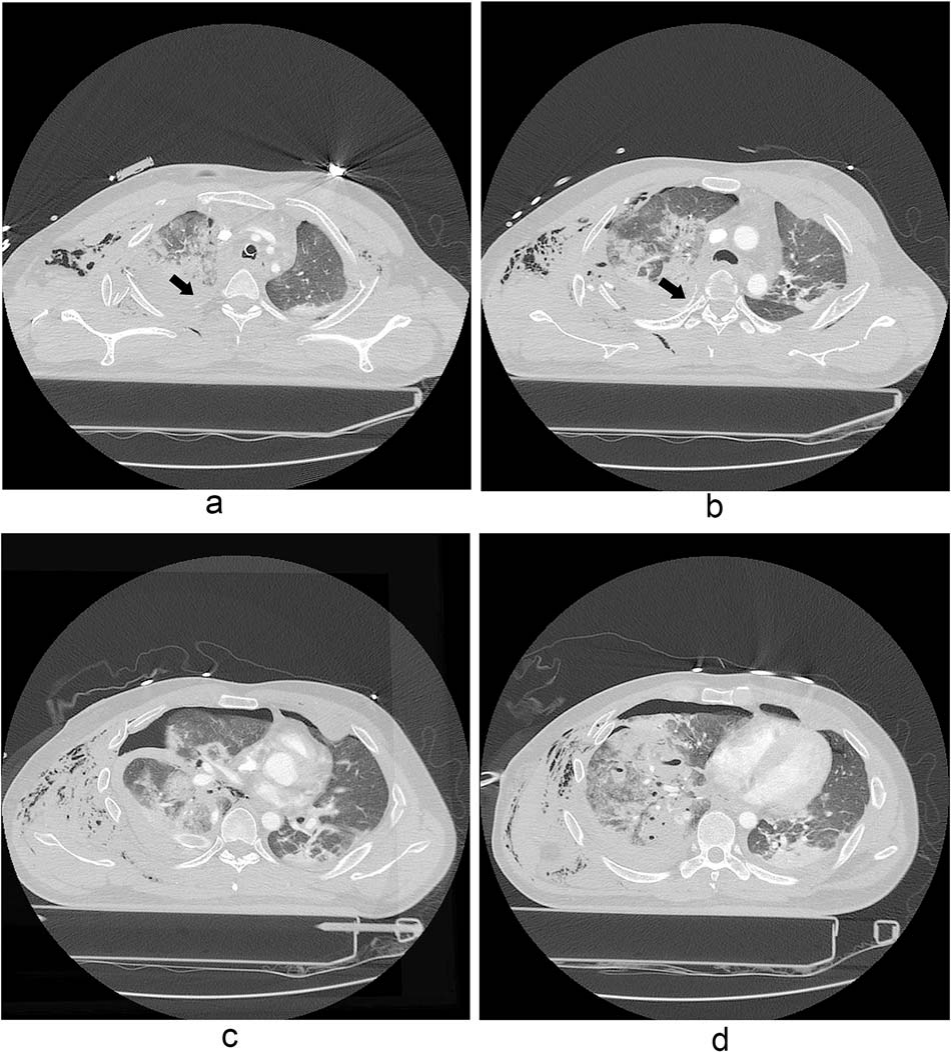
A contrast-enhanced chest CT scan after blunt chest trauma. Chest radiograph showed right hemopneumothorax and left hydropneumothorax. Multifocal extensive hemorrhage/pneumatocele formation was visible on the right side along with combined active bleeding (arrows) in the right lung.

A 31-year-old unconscious male was in an unwitnessed accident when a car hit the motorcycle he was driving. A rescue team transported him to the emergency department. Clinical examination revealed hypoxia with SpO_2_ at 85%, tachycardia at 126 bpm, hypotension of 69/58 mmHg, and hypothermia at 35.8°C. Initial arterial blood gas analysis (ABGA), pH, PaO_2_, and pCO_2_ were 6.92, 49, and 58, respectively. Chest X-ray showed right hemopneumothorax, mediastinal shifting, and multiple bilateral rib fractures (Fig. 1). A right-side closed thoracostomy was performed. The chest drainage of 1 L was bloody. Chest computed tomography (CT) showed fractures in the right ribs 1 to 9 and left ribs 1 to 7, as well as lung contusions, multiple lung lacerations, right hemopneumothorax, and active hemorrhage in the right upper and lower lung lobes (Fig. 2). Brain and abdominopelvic CT showed no organ injury. An emergency thoracotomy was ordered.

**Figure 3:**
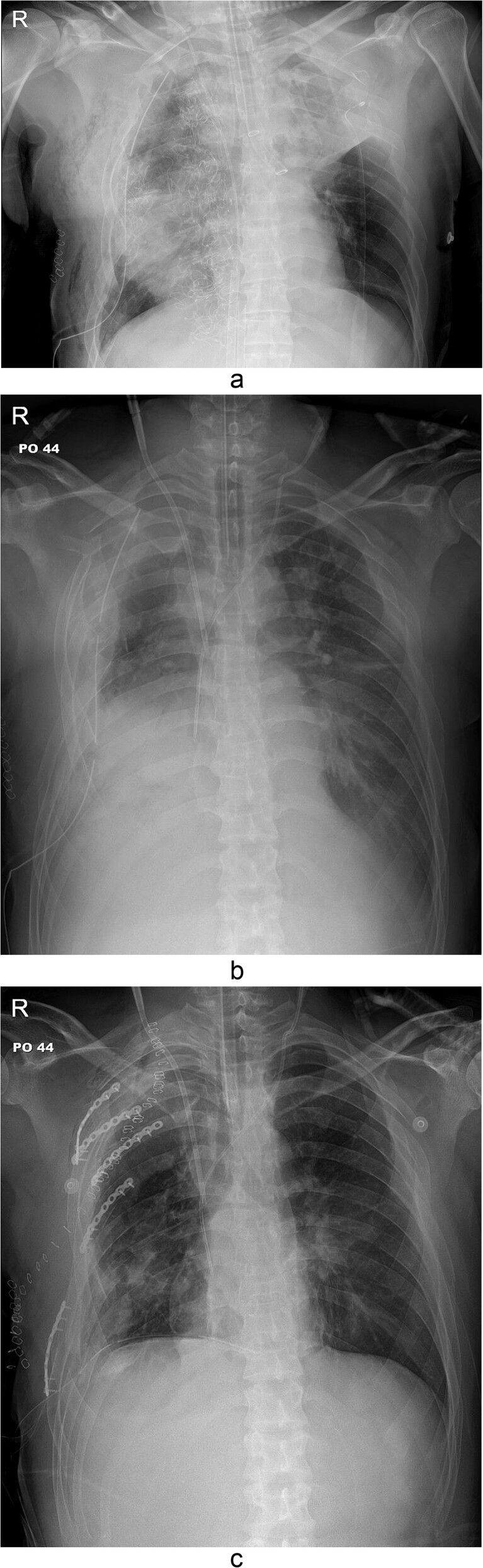
Simple radiographs obtained after each operation. (a) Radiograph after the first operation. Gauze, which is visible as a wavy white line, was packed in the right thoracic cavity. Multifocal opacity was seen in the right lung, and there was left upper lung atelectasis. ECMO catheters were placed in the IVC. (b) Radiograph following the second operation. The right ribcage is narrowed due to displaced fractured ribs. (c) Radiograph after the third operation. Five plates were applied to the fractured ribs.

**Figure 4:**
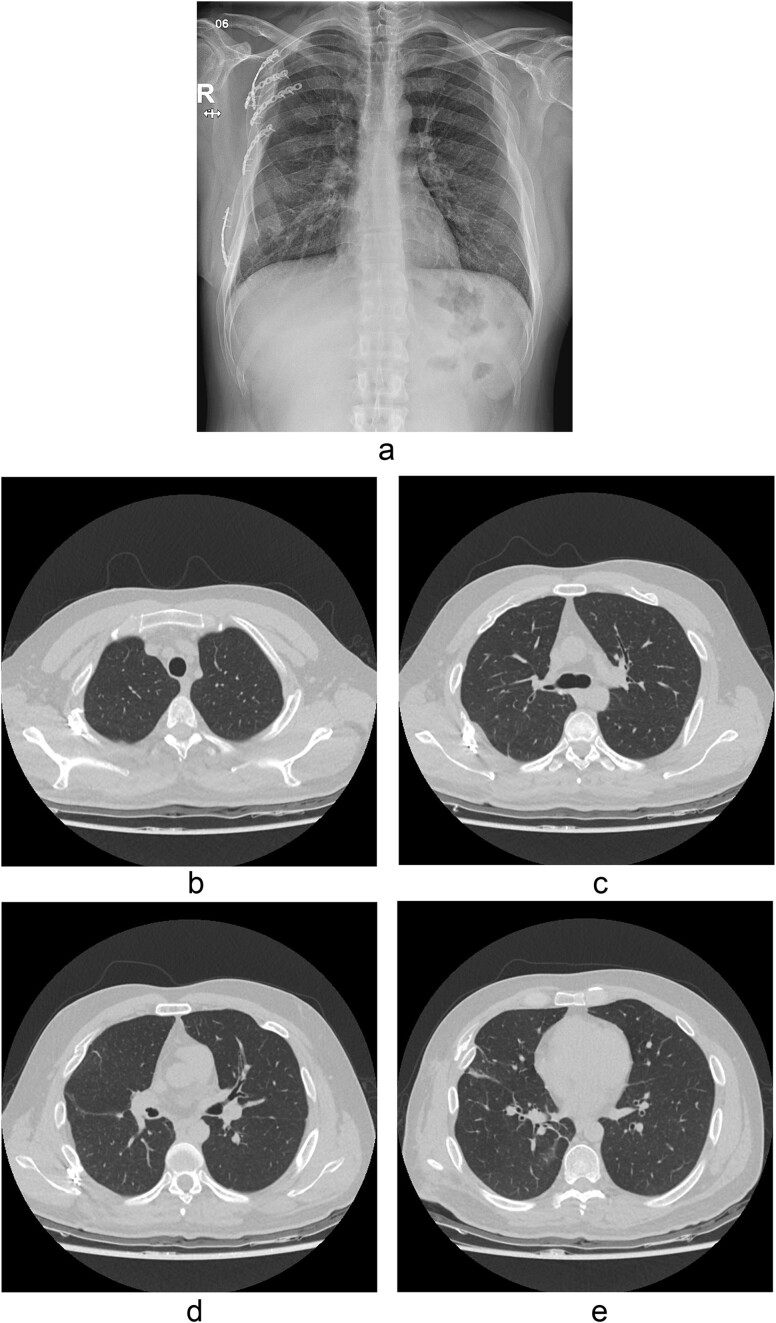
Chest radiograph and CT scan 10 months after the accident. (a) Chest radiograph showed well-healed fractured ribs and expanded lungs without active lesions. (b)–(e) Non-enhanced axial chest CT images showed recovery from injury.

The thoracotomy incision was relatively small, ~8 cm in size, and the intercostal space was not spread to prevent further intraoperative chest wall damage. There were multiple lung lacerations in the upper, middle, and lower lobes with active bleeding. Bloody leakage continued through the avulsed parietal pleura and mediastinum. Hemostasis was achieved by suturing the deeply lacerated lung and packing with gauze (Combat Gauze ™ [Z-Medica QuikClot]) at the chest wall and mediastinum. The operation took 130 minutes. The transfusion of plasma, platelets, and red blood cells was balanced according to the following quantities: 10, 10, and 14 U, respectively. After surgery, the patient was transferred to the intensive care unit (ICU). The PaO_2_/FiO_2_ ratio was 52 mmHg with a Positive end expiratory pressure of 15 cm H_2_O, at a peak inspiration pressure of 30 cmH_2_O. ABGA showed pH 7.23, pCO_2_ 42, and pO_2_ 47. Veno-venous ECMO based on bilateral femoral vein cannulation was performed (Fig. 3a). Considering the patient’s hemorrhagic predisposition, heparin was not used. Disseminated intravascular coagulation (DIC) occurred along with acute renal failure. Antithrombin III infusion and continuous renal replacement therapy (CRRT) were initiated on the second hospital day. By the 5th day, oxygenation was achieved without ECMO support, followed by decannulation. On the 6th day, the second operation was performed in about 35 minutes. The gauze was removed, and no active bleeding or oozing was seen (Fig. 3b). The third operation, performed on the 13th day, was an open reduction of right ribs 3–7 for correction of flail chest and took 75 minutes (Fig. 3c). After open reduction, the flail chest improved, but the patient was not able to be weaned off the ventilator. Pneumonia and bacteremia developed. A tracheostomy was performed on the 15th day. CRRT was switched to hemodialysis, which was stopped on Day 28. The patient was discharged on the 47th day without tracheostomy. Ten months postoperatively, pulmonary function testing revealed forced expiratory volume in one second and diffusion capacity of carbon monoxide of 77% and 67% of predicted values, respectively. The chest CT and radiographs showed that the lungs had healed well, and there was no displacement of the ribs (Fig. 4). The patient recovered and returned to work.

## Discussion

DCS is a staged surgical approach to manage critically injured trauma patients. It delays definitive surgical treatment in the ICU until normal physiology is restored [1].

In this case, extensive intrathoracic damage and a bleeding tendency were observed. The bleeding due to contusion injuries to the chest wall and mediastinum was not managed by conventional methods and was addressed with gauze packing. Moriwaki [2] suggested the indications for gauze packing in DCS as follows: (i) hemorrhagic shock owing to intrathoracic bleeding that requires an emergency thoracotomy, (ii) severe acidosis according to ABGA, macroscopic coagulopathy, and hypothermia in operative findings during thoracotomy (lethal triad), and (iii) no other way to stop the bleeding in a short time.

In this patient, Combat Gauze, named because it was used by United States Army soldiers, instead of regular gauze was used to stop bleeding. The Combat Gauze pad contains a hemostatic agent and has been reported to reduce bleeding and death in femoral artery and liver swine injury models [3]. Choron *et al.* [4] reported the safety of a laparotomy pad and QuikClot (Combat Gauze or Trauma Pad) gauze to control bleeding in DCS.

The use of ECMO in patients with traumatic lung injury has the potential to cause bleeding. The systemic anticoagulation required for ECMO may be contraindicated because it could promote further bleeding. Nevertheless, there are cases in which ECMO must be applied due to uncontrollable respiratory failure during ventilator care. In the present case, a severe bleeding predisposition and thrombocytopenia were observed, and heparin was not used for ECMO. Instead, transfusion of fresh frozen plasma and platelet concentrate was required until the fourth day of hospitalization due to the accompanying DIC and bleeding tendency. The lowest platelet count per day was 71 000 on the first day and then ranged from 24 000 to 40 000 thereafter. On the 7th hospital day, the patient’s platelet count reached 72 000. Rie *et al.* [5] reported the results of ECMO in acute respiratory failure patients with severe chest trauma. Patients at high risk of additional bleeding or clear intracranial hemorrhage were advised not to use heparin within 48 hours until hemostasis was achieved.

In the present case, hemostasis was achieved using lung suturing, gauze packing, and blood transfusions. Use of ECMO without heparin anticoagulation allowed DCS so the patient could recover without any serious complications.

## References

[1] Manzano-Nunez R , Chica J, Gomez A, et al. The tenets of intrathoracic packing during damage control thoracic surgery for trauma patients: a systematic review. Eur J Trauma Emerg Surg 2021;47:423–34. 10.1007/s00068-020-01428-8. 32594214

[2] Moriwaki Y , Toyoda H, Harunari N, et al. Gauze packing as damage control for uncontrollable haemorrhage in severe thoracic trauma. Ann R Coff Surg Engl 2013;95:20–5. 10.1308/rcsann.2013.95.1.20. PMC3964630 23317720

[3] Arnaud F , Okada T, Solomon D, Haque A, Carroll EE, Sagini Eet al. Initial evaluation of a nano-engineered hemostatic agent in a severe vascular and organ hemorrhage swin model. J Truam 2012;73:1180–7. 10.1097/TA.0b013e31825b3a60 22914081

[4] Choron RL , Hazelton JP, Hunter K, et al. Intra-abdominal packing with laparotomy pads and WuikClot™ during damange control. Laparotomy:a safety analysis. Injury 2017;48:158–64. 10.1016/j.injury.2016.07.033. 27469399

[5] Ried M , Bin T, Philipp A, et al. Extracorporeal lung support in trauma patients with severe chest injury and acute lung failure: 1 10-year institutional experience. Crit Care 2013;17:R110. 10.1186/cc12782. 23786965 PMC4056791

